# The International Society of Nephrology Collaborative Quality Framework to Support Safe and Effective Dialysis Provision in Resource-Challenged Settings

**DOI:** 10.1016/j.ekir.2024.11.1366

**Published:** 2024-12-04

**Authors:** Simon Davies, Saraladevi Naicker, Adrian Liew, Tushar Vachharajani, Roberto Pecoits-Filho, Vivekanand Jha, Fredric Finkelstein, David C.H. Harris

**Affiliations:** 1 School of Medicine, Faculty of Medicine and Health Sciences, Keele University, Staffordshire, UK; 2 Department of Internal Medicine, Faculty of Health Sciences, University of the Witwatersrand, Johannesburg, South Africa; 3 Mount Elizabeth Novena Hospital, Singapore; 4 School of Medicine, Wayne State University, Detroit, Michigan, USA; 5 Pontificia Universidade Catolica do Parana, Curitiba, Brazil; 6 Arbor Research Collaborative for Health, Ann Arbor, Michigan, USA; 7 The George Institute for Global Health, University of New South Wales, New Delhi, India; 8 Manipal Academy of Higher Education, Manipal, India; 9 Faculty of Medicine, Imperial College London, London, UK; 10 Department of Medicine, Yale University, New Haven, Connecticut, USA; 11 Centre for Transplant and Renal Research, Westmead Institute for Medical Research, The University of Sydney, Sydney, New South Wales, Australia

**Keywords:** global health, health policy, hemodialysis, peritoneal dialysis, quality standards

## Abstract

Global underprovision of affordable dialysis results in inequitable access to safe and effective treatment. The need for a dialysis quality framework that would set minimum standards for safe and effective treatment, was identified as a key priority for the International Society of Nephrology (ISN) Kidney Failure Strategy. In addition, this framework, which was developed through collaboration and iterative review to ensure external validity, links these standards to resource requirements and suggested reporting tools. The framework includes elements that are designed to minimize catastrophic health care expenditure, by mandating transparent individualized affordable dialysis planning and encourages continuous quality improvement by including a tiered approach to standards. Although a set of minimum mandatory standards is included with a strong focus on patient safety, this is not intended as a goal for all, but to support the incremental development of dialysis in the most challenging resource settings. Providers and funders are expected to engage with the framework at a level commensurate with their resources, clearly identifying the area where lack of resource is leading to suboptimal quality. In this way, the framework empowers policy makers, health care commissioners, and patient groups to work with providers to ensure quality and meet demand.

### Introduction

There is a substantial underprovision of dialysis treatment for kidney failure worldwide. Conservative estimates suggested that more than 2.6 million people died without recourse to this life-saving kidney replacement treatment in 2010, mostly in lower resource settings, and this figure is projected to double by 2030.^1^ This is not surprising given the high costs involved. Many have argued that it is inappropriate for low-income countries to develop dialysis services for chronic kidney failure as a component of a universal health care policy, and it is better to spend available funds tackling risk factors for chronic kidney disease and preventing its progression.^2^ However, things are never that simple. Where health care needs exist, there will always be a demand for services, a market to exploit, and politicians and health care policymakers will need to respond. Given that dialysis is available but outside the ambit of universal health coverage, patients frequently end up paying for treatment “out of pocket” with potentially disastrous financial consequences for them and their families. Even in countries where profound poverty exists, evolving middle classes quite rightly demand access to expensive health care.^3^ These conditions lead to significant challenges. Health care professionals wishing to provide dialysis find it difficult to obtain resources to deliver good quality care and manage the ethical and moral dilemmas this entails.^4^ Conversely, there is the risk of a regulatory vacuum in which poor practice can exist, putting kidney failure patients and their families at risk of harm and catastrophic health care expenditure.^5^^,^^6^

In 2018, the ISN hosted a summit to discuss the global inequity of access to integrated kidney failure care.^7^ This was the first step in developing a strategic plan to address the many challenges faced by the global kidney failure community, identifying several cross-cutting themes, including lack of data, need for advocacy, workforce challenges, financing, and ethical concerns (Figure 1). The need for a specific dialysis action plan was agreed on, with focus on defining a quality standards framework that was fit for purpose in resource-challenged settings. Although in no way implying that poor dialysis quality is the sole preserve of resource-challenged settings, it is evident that there are concerning variations in patient survival in these circumstances,^8^ which become especially evident during periods of stress, such as the COVID-19 pandemic and during wars and conflicts.^9^^,^^10^ Lack of information systems often means that reliable data on patient outcomes are unavailable.^11^ It has been observed that affordability of dialysis is only one of the many barriers facing patients in low-resource settings, others being quality of care and access to reliable information.^12^ The strategic plan was further developed at a second, more focused summit,^13^ at which a number of thematic workstreams were established, including dialysis, transplantation, conservative kidney management, resources, and monitoring (the latter being the development of methods to capture data, conduct surveys, and support registry development).^14^ The Kidney Failure Strategy Dialysis Working Group developed a work plan for a quality framework that would include “minimally acceptable” but mandatory standards that could be used to support local and national audits and service development, thereby providing patients with a charter for safe and effective dialysis that can be overseen by country level regulators. The work plan would also provide a roadmap for progressive improvement in standards. This article reports on the subsequent development and presentation of this framework.

**Figure 1:**
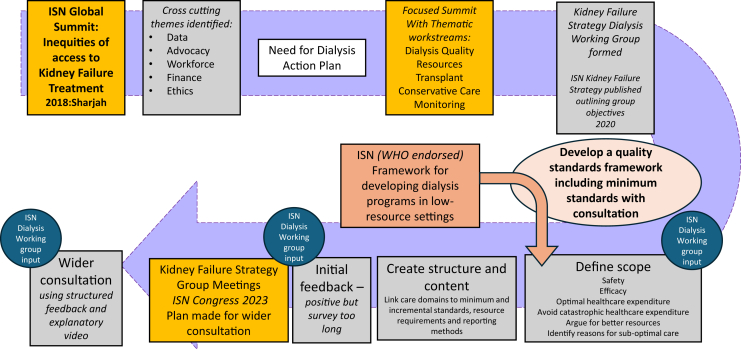
The sequence of steps leading to the development of the ISN Kidney Failure Strategy Dialysis Quality Framework is shown with meetings held at ISN Congresses in orange and key stages in the framework development are shown in grey with input from the ISN Dialysis Working Group indicated. The link with the ISN Framework for developing dialysis programs in low-resource settings is expanded in the text and Figure 2. ISN, International Society of Nephrology.

### Defining the Scope of the Framework

The Kidney Failure Strategy Dialysis Working Group met online during the COVID-19 pandemic and worked closely with the Resource Working Group and the ISN Dialysis Working Group^13^ because developing a framework that was not directly coupled to the required resources would not be helpful. It also drew on the recently published ISN framework for developing dialysis programs in low-resource settings,^15^ which was endorsed by the World Health Organization. That document provides the context for the current quality framework by describing the concept of an integrated kidney failure service, pointing out the barriers that will need to be overcome, describing the requirements for a dialysis service, and giving advice on how to set up a dialysis unit (Figure 2). It includes a chapter on the “Considerations for ensuring quality of dialysis”, suggesting potential quality domains that should be considered and includes an extensive survey of local guidelines developed for a variety of resource settings and a series of inventories of essential laboratory tests, medications, water treatment system requirements, and supplies required to run a dialysis unit. However, while recognizing the need for safe and minimum standards this earlier document does not propose such a framework, but recommends that existing guidelines should be adapted to local circumstances (e.g., using the ADAPTE tool). Therefore, that guidance served as a valuable literature survey and a starting point when considering which quality domains should be included, and how they should be linked to resources and integrated into this new overarching framework that can then be used as a “call to action” by the global kidney failure community.

**Figure 2:**
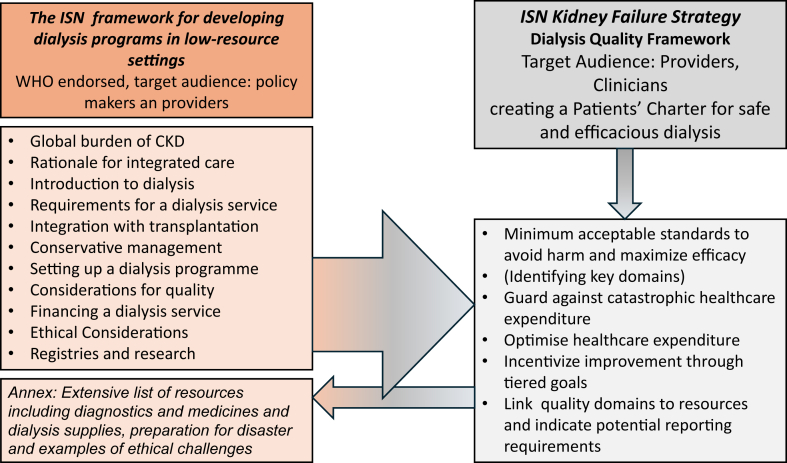
The relationship between the ISN Developing Dialysis and Kidney Failure Dialysis Quality Frameworks is shown. Whereas the Developing Dialysis framework is overarching and mainly aimed at policymakers, the Dialysis Quality Framework picks up on the considerations for quality and is intended as a charter for patients to get safe and effective treatment. ISN, International Society of Nephrology.

Early discussion focused on the scope, principles, and goals of the framework. In addition to the primary purposes of ensuring safety and maximizing efficacy with the resources available, several other goals were identified (Figure 1). These included components that would guard against catastrophic health care expenditure, enable health care providers to argue for better resources, optimize health care expenditure, and provide a structure that would incentivize improvement as discussed in the second summit. In addition, it was important that the framework should be encouraging rather than punitive in character and allow dialysis providers to distinguish between suboptimal care that was a consequence of resource limitation beyond their control, as opposed to that resulting from poor quality of delivered care. Such a recognition would allow the stakeholder community to generate demand for resources that support quality improvement. For example, twice-weekly dialysis may be recognized as inadequate for an individual but can be all that can be afforded; thus in this context, better than no dialysis at all. Conversely, dialyzer reuse is often deployed justifiably to save costs but when done to a poor standard, will be inefficient and potentially harmful. This is not to say that poor dialysis because of resource limitation is excusable; however, it is important that the cause for less than adequate care is identified so that appropriate remedial measures are taken. In situations where safety is a concern because of a failure to meet minimum safety standards, regulators should consider closure of the facility. It is also important to recognize that poor quality practice is a concern across the whole spectrum of resource settings, not just those that are very resource-challenged.

It was also important to clarify what the framework is not, that is, it is neither a replacement for existing guidelines adapted for use in every jurisdiction, nor is it a textbook of dialysis medicine designed to address clinical knowledge related to dialysis provision. Rather, it is intended as a framework for local guidelines and appropriate but robust audit measures.

### Framework Structure and Content

The resulting framework was structured in such a way that the proposed standards for each domain of dialysis care are directly linked to the resources and competencies required to deliver them alongside suggested reporting tools that should be used to audit adherence. Both hemodialysis (HD) and peritoneal dialysis are included in the same framework, which is divided into 2 main sections, namely Avoidance of Harm (Table 1) and Efficacy and Quality of Dialysis Care (Table 2). For each treatment domain, there are generally 3 levels of standards (occasionally fewer.) The first tier, denoted by ^a^ (see footnote to Table 1), represents an absolute minimum standard and is thus considered mandatory. Dialysis units, preferably during setup as well as when operational, should be able to demonstrate to regulators they are compliant. To encourage and support improvement in quality, more advanced standards are given, which dialysis providers should aspire to and use as leverage to obtain more resources.

**Table 1:** ISN Quality Standards Framework Section 1: Avoidance of Harm

Dialysis domain	Tiered standards	Reporting tools	Resources and competencies
Avoidance of Access Related Infection			
PD catheter–related infection (peritonitis and exit site infection)	1. Able to diagnose and treat, report cases and outcomes^a^ 2. Additional prevention measures in place (e.g., repeat training, exit-site prophylaxis, QA processes) 3. Work toward meeting ISPD standard (e.g., for peritonitis 0.4 episodes/yr, > 80% primary cure rate)	• Laboratory certification, if available^a^ • Local staff training certificates^a^ • Local audit • Regional/national reporting structure (cases, cure rate) • Sustainable antibiotic supply contracts	• Access to microbiology laboratory and antibiotics; staff trained to treat infections and train patients^a^ • Sufficient nurses to retrain, access to prophylactic antibiotics, team with QA training and experience • QI programs in place
HD access–related infection (fistulas or central lines)	1. Able to diagnose and manage access-related sepsis, report cases and outcomes^a^ 2. Prevention measures in place (access service, training in access care, supported by QA) 3. Work toward meeting an international standard (< 0.11 episodes/1000 patient days)	• Laboratory certification, if available^a^ • Local staff training certificates^a^ • Local audit • Regional/national reporting structure (cases, cure rate) • Sustainable antibiotic supply contracts	• Access to pathology laboratory and antibiotics; staff trained to manage access and treat infections^a^ • Staff trained in prevention measures, regular implementation • QI programs in place
HD water purity standard	1. A functioning and well-maintained system for water purification and dialysate reconstitution is essential in line with AAMI guidance^28^^,^^a^ 2. Meet international minimum standard [ISO 13959:2014 bacterial count of <100 CFU/ml and endotoxin concentration of <0.25 EU/ml]^16^ 3. Meet ultrapure standard [bacterial count of <0.1 CFU/ml and endotoxin concentration of <0.03 IU/ml]^17^	• Certification (e.g., public health laboratories, if available)^a^ • Regular audit (monthly) of results of water testing, e.g. bacterial and endotoxin contamination	• A secure water supply is essential. Water purification plant and a reliable source of dialysate concentrates.^a^ • Trained laboratory and technical support to undertake regular testing of water (or a system in place to send samples to a certified laboratory) and financial resources to undertake the necessary surveillance^b^
Dialysis equipment maintenance	1. Embedded plan for dialysis equipment maintenance (delivered by local trained staff or equipment suppliers)^a^	• Certification of trained technicians^a^ • Contracts with dialysis technology suppliers include ongoing plans for maintenance and training^a^ • Maintenance of an equipment register	• Technical support available to ensure dialysis equipment is maintained and/or repaired in case of failure, including a dedicated area. Sustainable access to essential equipment parts^a^
HD dialyzer reuse	1. Basic standards of safety must be used, in line with AAMI guidance^18^^,^^a^	• Systems for dialyzer labelling • Routine reporting and monitoring of reuse reaction^a^ • Systems to report testing of dialyzer function. E.g., check urea reduction ratio, visual inspection for thrombosis	• Equipment available for testing dialyzers before reuse and staff are trained to use this^a^ • Maintenance of automated systems for dialyzer reuse.
Avoidance of blood and airborne virus or bacterial infection	1. Systems in place to avoid blood contamination and reporting of cases to public health^a^ 2. Dialysis unit has proactive vaccination and surveillance of infection for staff and patients with ability to isolate infected individuals 3. Demonstration of virus-free or stable unit with no intraunit spread of cases. 100% staff vaccinated and antiviral treatments used where indicated	• Laboratory certification^a^ • Regional/national reporting structure (public health surveillance) • Annual audit report that includes new cases, proportions vaccinated and completeness of surveillance program	• Access to bacteriology and virology laboratory facilities to screen for hepatitis B, C, and HIV. All staff trained in basic infection control to avoid direct air- or blood-borne contamination^a^ • Able to undertake regular serology testing; sufficient dialysis stations to enable isolation of infected patients. Availability of vaccines and antiviral medication. • Routine audit and reporting processes in place
Safety Protocols for dialysis procedures and adequate staffing	1. Standard Operating Procedures (SOP) available and accessible for all procedures and sufficient staff to administer these (e.g., line and fistula management, catheter insertion, dialysis procedures – e.g., needling, cleaning of equipment between shifts)^a^ 2. Demonstration of regular audit of SOP compliance	• Demonstration of an accessible SOP repository^a^ • Staff training certificates^a^ • Audit of sufficient staffing to undertake safe dialysis – e.g., numbers of patients per dialysis nurse or health care assistant or technician. One person per 4–5 HD and 20 PD patients, depending on local role definitions	All staff undertaking dialysis-related procedures should be taught and assessed in their competencies or otherwise supervised by trained staff. There should be sufficient staff numbers (nurses, health care assistants, technicians, and access to regular physician review) to deliver the dialysis treatment safely^a^
Preparedness for catastrophic service failure	1. Developed disaster plan according to local risk assessment and resources^a^	• Documentation^a^	· Capacity to undertake a risk assessment of potential causes of service disruption, e.g., loss of power or water supply^a^

AAMI, Association for the Advancement of Medical Instrumentation; HD, hemodialysis; ISN, International Society of Nephrology; ISPD, International Society for Peritoneal Dialysis; PD, peritoneal dialysis; QA, quality assurance; QI, quality improvement.

The framework considers each domain of dialysis in terms of the standards to be met, proposed reporting tools and the resources and competencies required to deliver them. The numbers reflect the proposed tiers in ascending order of quality. The framework should be adopted as a charter for patients and their families and so made available along with transparent reporting of the dialysis units’ performance.

a The first level represents a mandatory minimum standard, (i.e., for centers with very low-resources) are indicated by ^a^; however, the standard that a dialysis center aims for should not default to the minimum standard but reflect their available resource. The resources and competencies do not directly correspond to the standards; however, the resources required to achieve the minimum are denoted by ^a^.

b This is controversial, but reflects the fact than many centers do not have access to specialist laboratories and in particular endotoxin testing (see discussion section for further detail).

**Table 2:** ISN Quality Standards Framework Section 2: Efficacy and Quality of Dialysis Care

Dialysis domain	Tiered standards	Reporting tools	Resources and competencies
Individualized sustainable dialysis planning	1. Where resources limit choice or options, this is made explicit; (pre- or dialysis plans include discussion of financial sustainability and “out of pocket” expenses. All modality options should be discussed, including transplantation, dialysis, and supportive care (which should be available, whether by choice or choice restricted).^a^ 2. Where resources allow, decisions are supported by shared decision making	• Report proportion of patients on transplant waiting list and audited^a^ • Report availability of dialysis modalities (e.g., is PD available, is it compulsory PD first)^a^ • Report actual use of modalities (dialysis, transplantation, supportive care, both by choice and choice restricted)^a^ • Report proportion of patients unable to have dialysis because of resource limitations including detail on sources of funding, part funding and what funding covers – e.g., drugs, EPO etc.**^a^** •Staff training certificates in Shared Decision Making	• Staff trained in establishing and documenting reasons for resource limitation and problem solving where resources are limited.^a^ • Staff should be trained to be able to have appropriate discussions about individualized prognosis and how this is affected by different treatment options^a^ • Staff trained in Shared Decision Making and unconscious bias
Dialysis quantity			
Hemodialysis	1. Less than standard care (e.g., <x3/wk for anuric patients) because of resource limitations, with reasons audited and recognized as sub-optimal^a^ 2. Able to provide standard HD care to all patients accepted for dialysis 3. Able to increase dialysis beyond standard care (extra sessions) if clinically required	• Report numbers or % of people having less than standard care for resource reasons^a^ • Specify reasons for resource limited care, e.g., lack of patient funds or lack of facilities^a^ • Report more than standard dialysis	• Provision of basic dialysis equipment, including consumables sufficient to sustain life. Sufficient trained staff to deliver dialysis (see also under avoiding harm)^a^ • Sufficient dialysis stations to provide dialysis with spare capacity for emergency treatments • Spare dialysis capacity
Peritoneal Dialysis	1. Less than standard care (e.g., daily CAPD x3–4 exchanges for anuric patients) for resource reasons; audited and recognized as suboptimal^a^ 2. Able to provide standard care 3. Encouraged to consider APD, dialysis assistance if clinically indicated	• Report numbers or % of people having less than standard care for resource reasons^a^ • Specify causes of resource limitation e.g., lack of patient funds or lack of facilities • Report proportions using APD, specialized solutions, dialysis assistance	• Stable access to supply of PD fluids (basic 2 L bags) sufficient to sustain life^a^ • No dialysis supply limitation • Access to assisted or automated PD and specialized PD fluids if practical and available
Monitoring processes	1. Documentation of dialysis attendance or access to PD fluid and urine volume.^a^ 2. Regular (monthly) monitoring of blood (Hb, K, Urea (pre-post dialysis), CO2, Ca, pH) and PTH – (3 monthly) is available. 3. Monthly QA sessions in place	• Audit survival on dialysis and hospitalization rates^a^ • Report numbers of sessions lost/patient • Laboratory certification • Regional or national reporting structure	• Maintenance of databases of dialysis delivery. Knowledge of residual kidney function.^a^ • Access to pathology labs and adequate resources to undertake regular predialysis bloods and urea reduction ratio and/or Kt/V measurements • Regional/national reporting structure
Potassium	1. Known episodes of hyperkalemia documented and audited. Able to give dietetic advice.^a^ 2. Undertake routine dietetic support and dialysis frequency avoidance of hyperkalemia (K > 6) in 90% of patients	• Local and regional reporting of K data^a^ • Report to regional/national registry	• Staff trained in basic hyperkalemia prevention. Able to measure K on demand^a^ • Routine monitoring (see monitoring processes) and access to trained dietitian (See dietetic service) • Regional/national reporting structure • Utilization of K binders if available
Acidosis	1. Where limitation of dialysis quantity does not control acidosis, oral bicarbonate supplements are available and used^a^ 2. Sufficient dialysis available to control acidosis	• Local audit of bicarbonate data^a^	• Able to measure bicarbonate^a^ • Supplement dialysis of needed with oral bicarbonate if dialysis availability restricted
Mineral metabolism	1. Routine monitoring includes measures of mineral metabolism^a^ 2. Audit: Aim for >80% of patients with PO4 <2.5 mmol/l 3. Prevention of severe -PTH	• Report availability of PO4 binders, Vit D^a^ • Local and regional reporting of Ca++, PO4, and PTH data	• Availability of PO4 binders and Vit D supplements to manage mineral metabolism^a^ • (See *dietetic service section* for staff training and competencies) • Surgical or medical treatment of severe -PTH available
Anemia management	1. Correction of iron deficiency by iron supplements; where available, EPO is used^a^ 2. EPO universally available 3. Audit: 85% of patients with 100 > Hb < 115 units?	• Report availability of iron and EPO^a^ • Local and regional reporting of anemia management data	• Able to prescribe iron supplementation (oral, i.v.) and monitor Hb response^a^ • EPO available; systems for monitoring EPO management (Laboratory: Ferritin, TSats; • Team QA meetings and QI expertise; Regional/national reporting structure
Blood pressure and volume management	1. Measurement of BP and documentation of episodes of fluid overload. Routine assessment of BP and fluid status in place and audited^a^ 2. Prevention of fluid overload with individualized treatment regimens, avoiding excessive UF rates (i.e., < 10 ml/kg/h on HD)	• Regional/national reporting structure for BP, interdialytic weight gains and UF rates^a^	• Blood pressure measuring and weigh scales equipment available; staff trained to undertake measurements^a^ • Access to high BP medications^a^ • Education pf staff and patients in fluid management (see dietetic services)
Access service	1. Service that enables the initiation of dialysis in a timely fashion, audit of access complications^a^ 2. Permanent fistula in HD or PD catheter access is the goal 3. >66% of dialysis patients with permanent access	• Local audit of access, to include % with functioning access^a^	• Access to team to establish urgent dialysis access (lines/PD tubes); availability of equipment, lines^a^ • Dedicated staff for access creation (PD and HD) • Access QA program in place with assistance of an access coordinator
Dietetic service	1. Staff have basic training in dietetic requirements in kidney failure^a^ 2. Dietetic led service with access to renal trained dietitian if needed 3. Regular prospective review of dialysis patients by renal dietitian	• Local audit of level of service delivery^a^ • Staff training certificates to demonstrate basic and higher-level competencies	• All staff have basic training in the dietetic requirements of kidney failure (e.g., K, PO4, fluid intake)^a^ • Dietetic literature developed for local needs and overseen by a trained dietitian. Availability of nutritional supplements • Dietetic support provided by trained renal dietitians with capacity for pre-emptive management

APD, ambulatory PD; BP, blood pressure; CAPD, continuous ambulatory PD; Ca, calcium; C02, carbon dioxide; EPO, erythropoietin; Hb, hemoglobin; HD, hemodialysis; ISN, International Society of Nephrology; ISPD, International Society for Peritoneal Dialysis; K, potassium; PD, peritoneal dialysis; PO4, phosphate; PTH, parathyroid hormone; QA, quality assurance; QI, quality improvement; UF, ultrafiltration.

For explanation see legend and footnotes for Table 1.

Following discussions with the ISN Dialysis Working Group, it was recognized that there is an inherent tension between setting minimum quality standards that would be unachievable, thus inhibiting any development of dialysis provision in very low-resource settings and standards that were so low that policymakers and payers could use the framework to reduce resources to a minimum, and providers could use it as a justification for low-quality treatment. The expectation is that policymakers, health care professionals, and dialysis providers would work together to use this framework in the context of their own resource setting, For example, new services in very low-income countries or regions might predominantly aim initially for tier 1 (mandatory minimum), whereas those in low-to-middle–income, and even high-income countries, many of which for example, do not have open discussions about dialysis modality choice, would aim to deliver tier 2 standards or higher. Any regions that do not have services conforming to the highest standard would engage in a process of iterative improvement as resource availability improves. By making the local performance to the framework publicly available, in effect as a “dialysis charter,” all stakeholders, not least patients, their families and carers, would be able to judge whether the level of standards achieved is appropriate for their resource setting. For each setting, the expectation was that the standards would gradually improve with improvement in resources.

To address the avoidance of catastrophic health care expenditure, the minimum standard is designed to document that individualized and sustainable planning for kidney failure replacement treatment has taken place. This would include a transparent discussion about financial sustainability, the implications for “out of pocket” expenses for the patient and their family, and the options for transplantation; for example, is dialysis limited to being a short-term bridge to living-related donor transplantation, or to meet a specific life goal? There should be honest and transparent discussions before commencing dialysis about what will happen if funding is no longer available. Health care systems should require dialysis centers to audit the use of all treatment modalities in their center, including transplantation, and to document the number of patients that cannot pursue a dialysis plan because of resource limitations.

With respect to the suboptimal provision of dialysis below the generally accepted standards of care (e.g., less than 3 sessions per week in the case of HD, or less than 3 or 4 continuous ambulatory peritoneal dialysis exchanges per day resulting in clinical evidence of suboptimal treatment that is not part of a planned incremental dialysis start), the reasons for this should be documented and monitored. Potential causes could include lack of funds, lack of facilities (which may include space and dialysis equipment), inadequate staffing or the patient’s inability to adhere to treatment (which might be affected by financial or nonfinancial factors outside their control). Remedial mechanisms should be explored where appropriate.

### Evaluating and Refining the Framework

An iterative process of obtaining feedback was undertaken to establish the external validity of the framework (Figure 1). Initially, this was from the ISN Dialysis Working Group (see the Acknowledgments) and a pilot online survey, sent to selected clinicians practicing in lower-resource settings. This initial survey was unduly long; thus, at a meeting of the ISN Kidney Failure Strategy Group at the World Congress of Nephrology in Bangkok in 2023, these pilot data and progress with the project was presented to those at the conference who attended the previous 2 summits. It was agreed to progress the project further, developing a second differently structured evaluation process (Supplementary Table S1) with survey questions reformulated to establish whether the framework was meeting its core objectives. Distribution of the survey was via the ISN, and this time accompanied by a short video presentation explaining how the framework was developed and intended to be used (Supplementary Material). This was sent out to all the attendees of the Bangkok discussion and individuals with selected expertise, for example, in ethics and health economics (see the Acknowledgments). Following this evaluation, further changes were made.

Formal written feedback was obtained from 31 individuals selected as experts in delivering dialysis in resource-challenged settings, including dialysis unit administrators and experts in dialysis technology, or with expertise in shaping kidney failure health policy (e.g., with the World Health Organization or health economics) from 19 countries, including Australia, Canada, China, Eswatini, India, Japan, Kenya, Malawi, Malaysia, Mauritius, Mexico, Pakistan, Russia, South Africa, Switzerland, Uganda, UK, United Arab Emirates, and USA. In addition, the framework was discussed at sequential meetings of the ISN Dialysis Working Group, with additional representation from Georgia and Senegal. For details of specific expertise, see the Acknowledgements. The following sections describe their feedback and how this was incorporated into the framework.

#### Safety Standards

Minor amendments were made in the section addressing infection control, including consideration of airborne infection, antiviral therapies, and vaccination. Greater emphasis was placed on maintaining a secure water supply, equipment maintenance, and dialyzer reuse. Preparedness for catastrophic service failure was added, recognizing that this risk is often most significant in low-resource settings.

#### Transparent Dialysis Planning

To address catastrophic health expenditure, there was universal agreement that this was one of the most important objectives of the framework and strong support for the approach based on transparent information and individualized, clearly documented sustainable planning. Clarification was requested, for example, explicitly including and differentiating between the options of supportive kidney care by choice or when this is choice-restricted. It should be emphasized that the framework shown in Tables 1 and 2 should be read with the explanatory notes, as described here in the text and the caption to the tables. Recommendations were also made that are already part of the ISN Kidney Failure Strategy,^7^^,^^14^ including strong advocacy for universal health care coverage and the need for research in low-cost dialysis equipment and solutions.

#### Efficacy Standards

The approach adopted in the framework was supported by most of the evaluations. Concerns were expressed related to recognition that less-than-standard dialysis regimens may be appropriate in some circumstances, for example, incremental dialysis or end of life. In terms of specific measures of dialysis efficacy (e.g., biochemical measures), the views were more split, recognizing that resource limitations might preclude regular monitoring, but that this is accounted for in the tiered system of standards.

#### Value of the Framework in Obtaining Resources

This feedback did not lead to many changes in the framework but picked up on particular aspects. For example, the framework makes much use of both audit and quality improvement, especially when attempting to move up the tiered standards. This requires investment in the workforce, leadership, training, expertise, and time for staff to complete the work involved, which is challenging. It was recognized that including this in the framework could help in demand generation and obtaining resources and that the ISN should continue to produce educational materials to support these activities. Feedback related to this is included in the Supplementary Material.

#### Appropriateness of Tiering

The tiered levels of the standards were generally considered to be conceptually helpful and appropriate. The exception was water purification, where the minimum standard originally set was considered unrepresentative of current practice in some low-resource settings. For example, some regions may not have regular access to endotoxin or bacterial testing to comply with current standards.

### Discussion

As with many clinical practice guidelines, those addressing quality standards for the delivery of dialysis address best practices in high-income countries. The current recommendation^19^ is that dialysis providers in resource-challenged settings should adapt these guidelines, for example using the ADAPTE framework,^20^ so that standards are set to deliver the best care possible. The problem with this approach is that the existing guidelines do not address many issues that are especially relevant in settings without universal health care coverage, a high level of catastrophic health care expenditure, and ethical and moral dilemmas facing those delivering health care.^4^ This framework is not intended to replace the existing guidelines, but represents the first comprehensive attempt to provide an overarching structure that acknowledges that access to high-quality care is not available to most people worldwide and that dialysis, even when not delivered to the highest possible standard, is a life-saving treatment that helps people achieve specific life goals. This required devising a minimum set of standards designed to secure a balance between the therapeutic benefits, and particularly, the avoidance of harm, with a realistic assessment of available resources.

It will be noted that the framework has steered clear of the concept of dialysis “adequacy” (i.e., targets for urea clearance), preferring to take a benchmarking approach of usual practice. In this, we have followed the lead taken by the International Society for Peritoneal Dialysis in defining dialysis quality in terms of treatment goals rather than surrogates such as Kt/V.^21^ In other words, the success of dialysis treatment is judged clinically, for example, maintaining adequate nutrition, satisfactory biochemistry, managing symptoms, avoiding hospital admissions, and ultimately patient survival. The clinician is then able to determine whether the dialysis is doing its job and, if judged insufficient, to record whether this is due to resource limitation and make recommendations for improvement.

One of the more contentious aspects was how prescriptive the framework should be. For example, clarity on appropriate staff-to-patient ratios was considered important, reflecting the concern that payers will always try to minimize these to reduce costs and/or there may be shortages of suitable trained staff, which is not an issue confined to resource-challenged settings. First and foremost, staff working in dialysis care (HD or peritoneal dialysis) must have the correct set of competencies to deliver safe care, as strongly emphasized in the framework. Second, it is difficult to be overly prescriptive at a global level with respect to precise ratios, given the substantial variations in how staffing roles are defined. Local definitions of a “patient-care” technician, health care assistant or a nurse, along with their seniority grade will differ, and evidence on the correct ratio is lacking,^22^ and that being too prescriptive might have unintended consequences.^23^ Furthermore, the case-mix will vary between and within units, with some centers encouraging varying degrees of self-care. However, some comments can be made. Most guidelines propose that during an HD shift, there should be at least 1 staff member for every 4 or 5 patients – although the specific job descriptions may vary a great deal. At least 1 senior person should be available on each shift, and there must be sufficient staff to undertake proper cleaning between dialysis shifts. For patients on peritoneal dialysis, the suggested equivalent would be 1 nurse per 20 patients; however, it should be recognized that this varies considerably. It can be as low as 1 per 100 patients with no clear association with patient outcomes,^24^ with huge variation in the definition of the role.^25^ Further work is required to define the roles and competencies required to support this framework, and this will be led by the Kidney Failure Strategy Resource Working Group.

Another example of how prescriptive (or not) the framework should be was the appropriate number of reuse cycles for dialysis membranes. This approach to reducing costs on a dialysis unit is well-established, and there is broad agreement that if done properly, reuse is safe and cost-effective.^26^ Our framework points users to adopt the practice described by the Association for the Advancement of Medical Instrumentation^18^ rather than specifying the number of reuses. However, this is done in some jurisdictions, for example, in Mexico the limit is 12, well below the documented number of reuses implemented in other countries such as Pakistan, where the average number in a safety and efficacy trial was reported as 47.^27^ The number of reuses at which cost savings can be made may well differ by country and region.

One especially difficult area fundamental to the quality of HD is water quality. Although there are clear guidelines to be followed,^16^, ^17^, ^28^ it is also clear that this can be challenging to achieve in very low-resource settings. Our feedback as well as the published literature indicate that international standards for water purity are frequently not being met^29^, ^30^, ^31^ and there is room for further research and development of water quality systems for low-resource settings. For this reason, the minimum guidance emphasizes ensuring a secure supply of water and chemical concentrates to manufacture dialysate and maintaining a functional water purification plant. Water management is also a major part of delivering environmentally friendly dialysis. Although this has not been incorporated specifically into this framework, there is recently published advice for low-resource settings.^32^

Undoubtedly, this proposed dialysis quality framework has strengths and weaknesses. The strengths include its formulation in the context of a wider kidney failure strategy and engagement with the broader global nephrology community during its development, facilitated by the ISN.^13^ The weaknesses include gaps in the evidence base and the need to accommodate tensions such as setting minimum targets that enable engagement in very low-resource settings without running the risk of inhibiting investment in higher quality services. It is worth reiterating that the expectation is that dialysis providers and policymakers will engage with the framework at a level appropriate for available resources, and regulators and patient groups will use it to hold providers to account. Although patients have not been directly involved in the formulation of the framework, they have had inputs in the wider ISN Kidney Failure Strategy. It is fully intended that patient groups will be approached during the dissemination phase. The hope is that dialysis centers will adopt the framework, which can act as a charter between users and providers, and following its dissemination the next step would be for the Kidney Failure Strategy Monitor Working Group to establish its uptake. Finally, it is important to emphasize that this framework should be considered as a work in progress, and thus subject to revision and feedback as it is implemented.

## Disclosure

SD has received lecture honoraria from Baxter Health Care and Fresenius Medical Care; was a member of the Ellen Medical Advisory Board, is a steering committee member for CSL Behring CSL300_2301 POSIBIL₆ESKD study, and is a trustee of Kidney Research UK. VJ has received honoraria from Bayer, AstraZeneca, Boehringer Ingelheim, GSK, Baxter Healthcare, NephroPlus, Chinook, Biocrust, Travere, and Zydus Biosciences, under the policy of all honoraria being paid to the organization. AL has received consulting fees and honoraria from 10.13039/100004702Baxter Healthcare, 10.13039/100015699Fresenius Medical Care, 10.13039/100006396Alexion Pharmaceuticals, 10.13039/100006400Alnylam Pharmaceuticals, 10.13039/100014931Arrowhead Pharmaceuticals, 10.13039/100014935BioCryst, Boehringer-Ingelheim, Chinook Therapeutics, Dimerix Limited, Eledon Pharmaceuticals, George Clinical, GSK, 10.13039/501100007132Otsuka Pharmaceutical, Vera Therapeutics, Visterra Inc, and ZaiLab Co Ltd. RP-F is an employee of Arbor Research Collaborative for Health, which receives global support for the ongoing DOPPS Programs (provided without restriction on publications by a variety of funders; for details see https://www.dopps.org/AboutUs/Support.aspx); and has received research grants from Fresenius Medical Care; consulting fees (paid to the employer) from 10.13039/100004325AstraZeneca, 10.13039/501100020141Akebia, 10.13039/501100004191Novo Nordisk and Fresenius, 10.13039/100004326Bayer, Boehringer, Novo Nordisk, and Akebia. All the other authors declared no competing interests.

## Supplementary Material

Supplementary File (PDF) **Table S1.** Template for feedback. Evaluators of the framework were asked to address the following questions. **Feedback on the use of the Framework for obtaining resources.**

## Footnotes

Supplementary File (PDF)
**Table S1.** Template for feedback. Evaluators of the framework were asked to address the following questions. **Feedback on the use of the Framework for obtaining resources**.
