# Supplementary material for: The International Society of Nephrology Collaborative Quality Framework to Support Safe and Effective Dialysis Provision in Resource-Challenged Settings
Source: Kidney Int Rep. 2024 Dec 4;10(3):663–72. doi: 10.1016/j.ekir.2024.11.1366 (PMC11993199; doi:10.1016/j.ekir.2024.11.1366)

The International Society of Nephrology collaborative quality framework to support safe and effective dialysis provision in resource-challenged settings.

Simon Davies, Saraladevi Naicker, Adrian Liew, Tushar Vachharajani,
Roberto Pecoits-Filho Vivekanand Jha, Fredric Finkelstein, David C.H.Harris

Supplementary Material

Supplementary Table 1: Template for feedback. Evaluators of the framework were asked to address the following questions:

Ensuring Safety: Are there important safety domains that are missing? Have we captured the essential resources required to deliver these?
Maximising Efficacy: Rather than specifying doses of dialysis this is framed as less than or equal to standard care and centres are expected to record reasons for using less than standard care (e.g., due to resource limitation). Is this approach useful? If not, why not?
Maximising Efficacy: Specific guidance on particular measures of dialysis efficacy (e.g. K+, acidosis, PO4) are included. Are these useful? Are the levels suggested appropriate?
Avoidance of catastrophic health-care expenditure: This is addressed by ensuring that individualised sustainable dialysis and transplantation planning takes place. Comments welcome.
Enable leverage of Resources: By including the resource requirements within the framework we hope that it can be used to obtain resources. Have important resources been left out? How would you use this framework to obtain resources?
Provide incentive for improvement: For each domain in the framework the standards have been tiered from 'minimal' acceptable to increasingly demanding. Are the tiers correct? Indicate if you would change any of them.
Additional comments and area of expertise

Feedback on the use of the Framework for obtaining resources:

"I would use this framework as a reference document to discuss with my country's policy-makers what is lacking in terms of the minimum internationally recommended standards of care and pointing out the more advanced tiers of quality standards that we have yet to attain i.e. benchmarking"

“There several LMIC that have no or outdated Renal Standards. It is important to continuously communicate and drive change with the local agency (Ministry and Department of Health) that are task with overseeing the implementation and revision of effective hemodialysis standards and requirements.”

“…some of our centers of hemodialysis are working on written policies and procedures through a public-private partnership. This framework will help to develop hemodialysis policies at the national level.”

ISN Kidney Failure Strategy

DIALYSIS and RESOURCES Groups

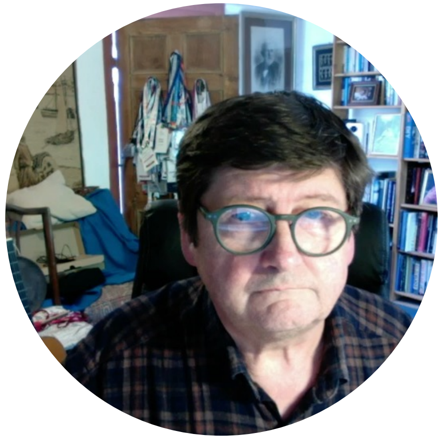

Strategic plan for integrated care of patients with kidney failure

Check for updates

David C.H. Harris[1,21], Simon J. Davies[2,21], Fredric O. Finkelstein[3,21], Vivekanand Jha[4,5,21], Aminu K. Bello[6], Mark Brown[7], Fergus J. Caskey[8,9,10], Jo-Ann Donner[11], Adrian Liew[12], Elmi Muller[13], Saraladevi Naicker[14], Philip J. O'Connell[15,16], Roberto Pecoits Filho[17,18] and Tushar Vachharajani[19]; on behalf of the Strategic Plan Working Groups[20]

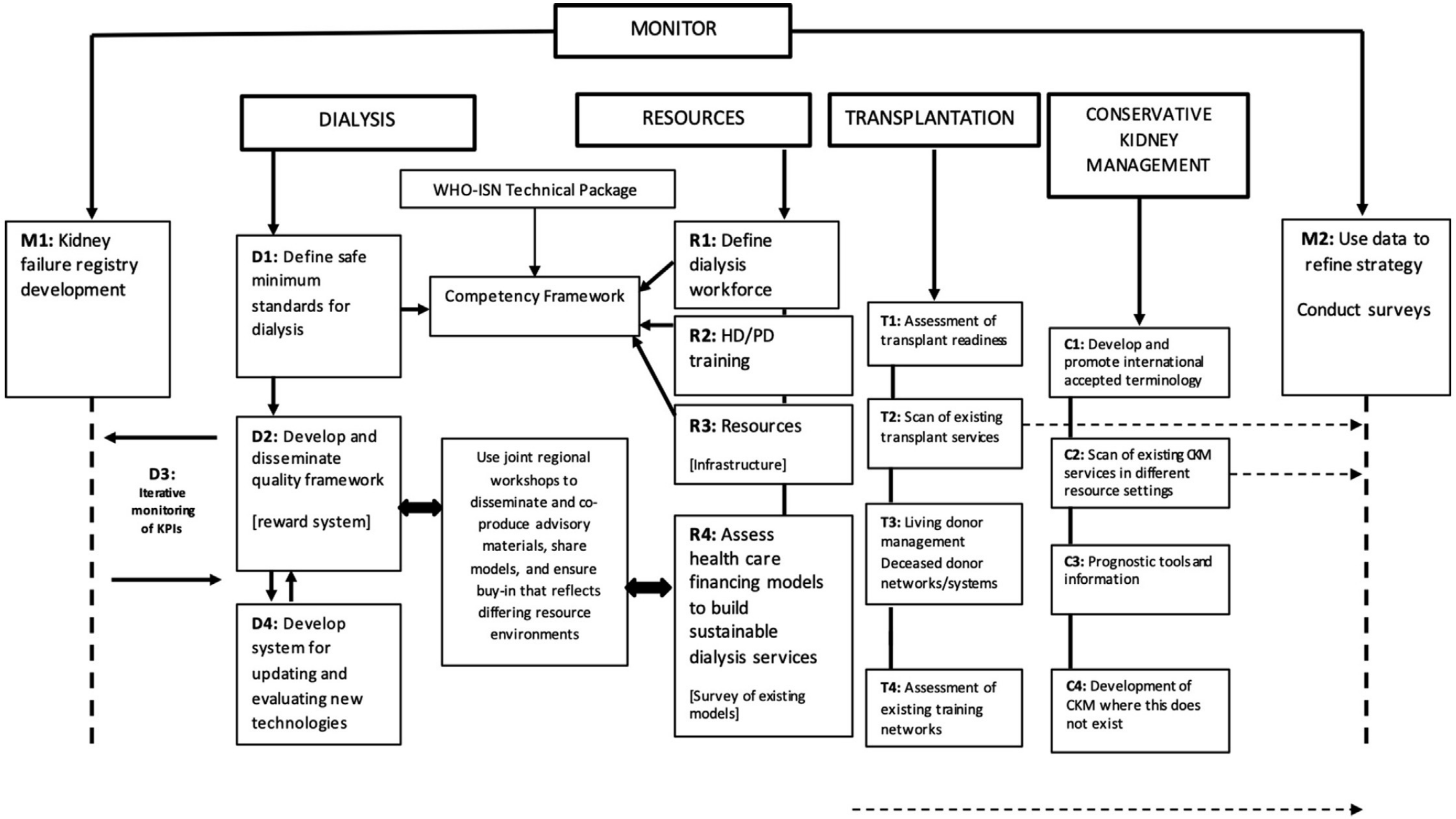

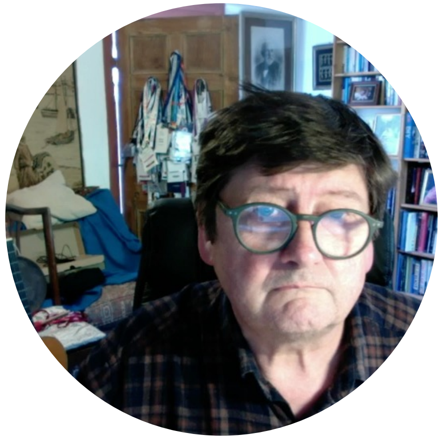

What does this quality framework try to do?

- Principles and Goals:
  - Ensure Safety
  - Maximise Efficacy
  - Address avoidance of catastrophic health-care expenditure
  - Enable leverage of resources
  - Optimise health-care expenditure
  - Provide incentive for improvement
  - Be encouraging rather than punitive
  - Distinguish between sub-optimal care due to resource limitation versu[illegible] quality of delivered care

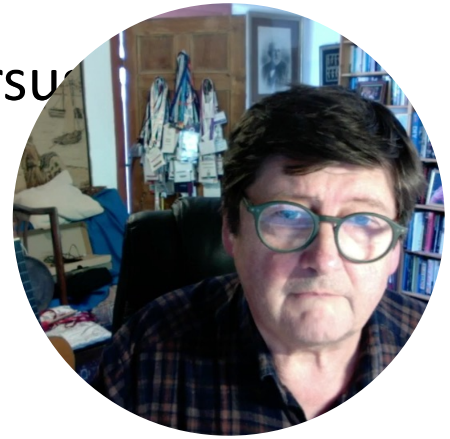

Quality Framework

Dialysis Domain (KPI)	Tiered Standards	*Reporting Tools*	Resources and Competencies
**SECTION 1: Avoidance of Harm**			
Avoidance of Access Related Infection			
**PD Catheter Related Infection**	1. Able to diagnose and treat, report cases and outcomes<br>2. Additional prevention measures in place (*e.g.* repeat training, ES prophylaxis, QA processes)<br>3. Work towards Meeting ISPD standard (*e.g.* 0.4 ep/year, >80% primary cure rate)	• Laboratory certification<br>• Staff Training Certificates<br>• Regional/national reporting structure (cases, cure rate)<br>• Sustainable antibiotic supply contracts	1. Access to microbiology laboratory and antibiotics; staff trained to treat and train patients<br>2. Sufficient nurses to retrain, access to prophylactic antibiotics, team with QI training and experience<br>3. Have QI programs [illegible]

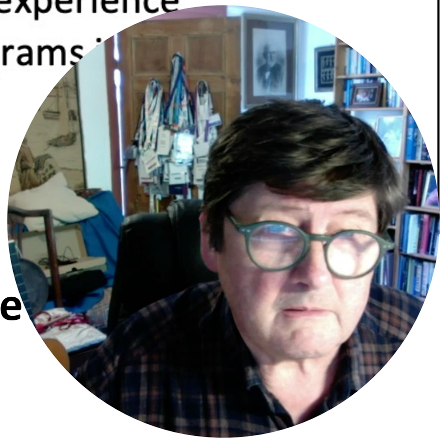

Level 1 = ‘Minimum’ acceptable Standard, Level 2 Level 3 = Goal (which is dictated by rele[illegible] body – itself a moving standard).

Dialysis Domain (KPI)	Tiered Standards	*Reporting Tools*	Resources and Competencies
**SECTION 2: Quality of Dialysis Care**			
Individualised sustainable dialysis Planning	1. Where resources limit choice/options this is made explicit; dialysis plans include discussion of financial sustainability and plans for transplantation<br>2. Where resources allow, decisions are supported by shared decision making	• Report proportion of patients on transplant waiting list<br>• Report availability of dialysis modalities (e.g. is PD available, is it compulsory PD first)<br>• Report actual use of modalities<br>• Report proportion of patients unable to have dialysis due to resource limitations<br>• Staff training certificates in Shared Decision Making	1. Staff trained in establishing and documenting reasons for resource limitation and problem solving where resources are limited<br>2. Staff trained in Shared Decision Making and unconscious bias
Dialysis Quantity			
**Peritoneal Dialysis**	1. Less than standard care (e.g. daily CAPD x3-4 exchanges for anuric patients) for resource reasons; audited and recognized as suboptimal<br>2. Able to provide standard care<br>3. encouraged to consider APD, dialysis assistance if clinically indicated	• Report numbers/(%) of people having less than standard care for resource reasons<br>• Specify causes of resource limitation<br>• Report proportions using APD, specialised solutions, dialysis assistance	1. Stable access to supple of PD fluids (basic 2 L bags) sufficien[illegible] life<br>2. No dialysis supply [illegible]<br>3. Access to assiste[illegible] and specialised PD[illegible] and available

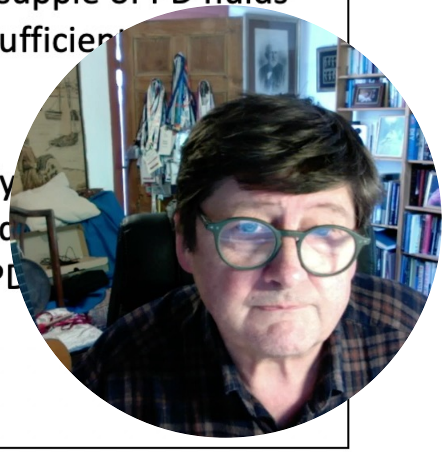

What we are asking you to do

ISN Kidney Failure Strategy DIALYSIS QUALITY FRAMEWORK Feedback Form

Once you have had a chance to look at the framework and watched the short explanatory video, please comment in the following aspects using free text:

Ensuring Safety: *Are there important safety domains that are missing? Have we captured the essential resources required to deliver these?*
Maximising Efficacy*: Rather than specifying doses of dialysis this is framed as less than or equal to standard care and centres are expected to record reasons for using less than standard care (e.g., due to resource limitation). Is this approach useful? If not, why not?*<br><br>*Specific guidance on particular measures of dialysis efficacy (e.g. K+, acidosis, PO4) are included. Are these useful? Are the levels suggested appropriate?*

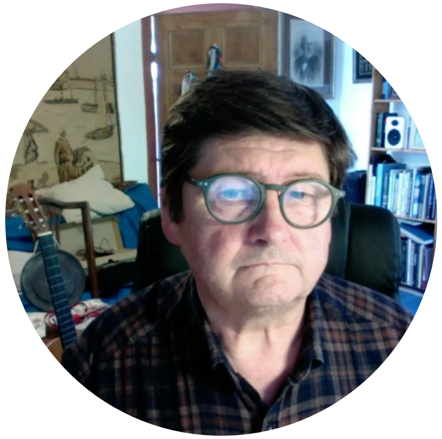

Thank you!

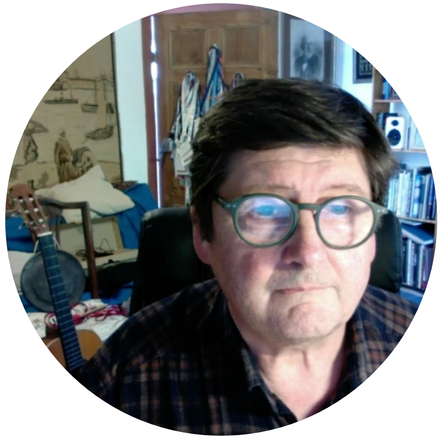

Supplement: Supplementary File (PDF) — Table S1. Template for feedback. Evaluators of the framework were asked to address the following questions. Feedback on the use of the Framework for obtaining resources. [file mmc1.pdf]
